# Maxillary mucocele in a 4-month infant

**DOI:** 10.1016/S1808-8694(15)30590-5

**Published:** 2015-10-19

**Authors:** Lucas Gomes Patrocinio, Priscila Garcia Damasceno, José Antonio Patrocinio

**Affiliations:** 1 Otorhinolaryngologist, physician of the otorhinolaryngology unit of the Uberlandia Federal University Medical School (Faculdade de Medicina da Universidade Federal de Uberlândia).; 2 Physician, resident in the otorhinolaryngology unit of the Uberlandia Federal University Medical School (Faculdade de Medicina da Universidade Federal de Uberlândia).; 3 Full professor, head of the otorhinolaryngology unit of the Uberlandia Federal University Medical School (Faculdade de Medicina da Universidade Federal de Uberlândia). Otorhinolaryngology unit of the Uberlandia Federal University Medical School (Faculdade de Medicina da Universidade Federal de Uberlândia), Uberlândia, MG, Brazil.

**Keywords:** mucocele, maxillary sinus, paranasal sinuses

## INTRODUCTION

Mucoceles are pseudocysts of the paranasal sinuses; they are lined with pseudostratified respiratory epithelium and have a mucous or mucopurulent (mucopyocele) content^1^, ^2^ Mucoceles are extremely rare in children, except in cases where there is a predisposition for obstruction, such as trauma, surgery, expanding lesions, chronic rhinosinusitis, allergy or cystic fibrosis.^1^, ^2^

The purpose of this paper is to report an extremely rare case of a maxillary mucocele in a lactating infant with no predisposing factors. Therapy was done with endoscopic surgery.

## CASE REPORT

**Figure 1:**
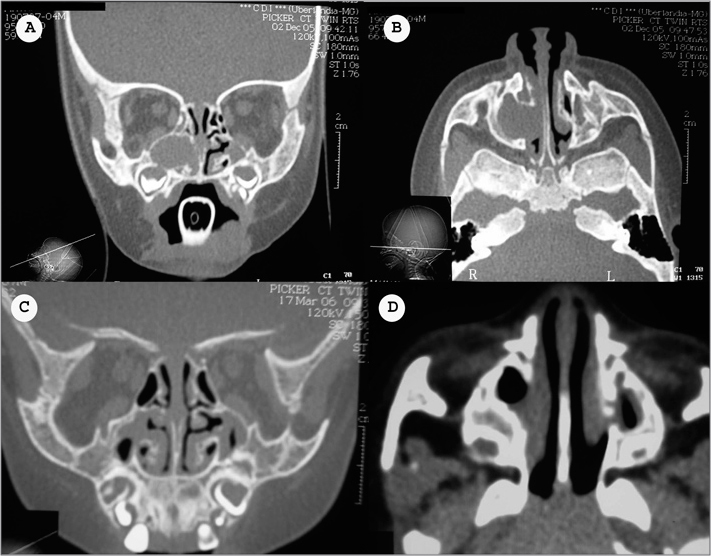
Computed tomography of the paranasal sinuses in axial and coronal sections showing a lesion with soft tissue density in the right maxillary sinus, bone erosion, and invasion of the ipsilateral nasal cavity (A and B). Eight months following surgery (C and D) there is edema of the mucosa in both maxillary sinuses, but no signs of recurrence of mucocele.

M.I.M.B., a female 4-month-old patient presented progressively worsening nasal block since birth. Videonasolaryngoscopy revealed secretion filling and obstructing the right nasal cavity, making it impossible to advance the optic fiber. Computed tomography (CT) of the paranasal sinuses showed a hyperdense mass in the right maxillary sinus, with medial bone loss and invasion of the ipsilateral nasal cavity, suggesting a mucocele (Figures 1A and 1B).

Endoscopic surgery was undertaken to remove the lesion. The specimen was sent to pathology, which confirmed the diagnosis of a mucocele. Videonasofibroscopy and CT were done eight months after the procedure, showing edema of the mucosa in both maxillary sinuses and no recurrence of the mucocele (Figures 1C and 1D). The patient has been followed up to the present date and remains asymptomatic.

## DISCUSSION

Mucoceles are pseudocysts lined with a respiratory mucosa that tend to grow slowly and expansively, leading to bone resorption of neighboring structures such as the orbit and the intracranial area. The prevalence is highest between the third and fourth decades of life; mucoceles are rare in children.^2^ Mucoceles are secondary to paranasal sinus surgery in 62% of cases, are primary in 35% of cases and are post-traumatic in 2% of cases. The most frequent site is the frontal sinus (60-65%), followed by the ethmoid sinus (20-30%), the maxillary sinuses (10%) and the sphenoidal sinuses (1%).^3^ We found no cases of mucoceles in infants aged 4 months - such as the present case - in the medical literature.

Clinical findings vary according to the site of the mucocele and whether there is involvement of adjacent structures.^4^ The etiology has not been well defined, but it is thought that mucoceles are related to factors that reduce mucous drainage, such as obstruction of ostia, chronic sinusitis, polyposis, trauma, prior surgery and cystic fibrosis.^1^

CT is the exam of choice for the diagnosis of mucoceles; it not only demonstrates sinus involvement, but also provides information about bone erosion and other effects on neighboring structures.^4^, ^5^

In this case, the mucocele was in the right maxillary sinus, it eroded the medial wall and invaded the nasal cavity, which explained the nasal block. Although maxillary sinus mucoceles are associated with prior surgery and - in children - with cystic fibrosis, the patient had none of these two factors.

Treatment is always surgical,^6^ which in this case was done by nasosinusal endoscopy. This approach should always be the option of choice, given its more physiological nature and its lower postoperative morbidity compared to external access procedures.^4^

Outpatient follow-up is mandatory to promptly identify recurrences.^5^ This patient is being monitored carefully, and has progressed favorably until now.

## CONCLUSION

Although extremely rare, mucoceles in children should not be neglected. Notwithstanding the benign nature of these lesions, expansion may cause severe intracranial and orbitary complications, particularly when these lesions are infected. Surgery is indicated in all cases.
